# Traditional cardiovascular risk factors and coronary collateral circulation: Protocol for a systematic review and meta-analysis of case-control studies

**DOI:** 10.1097/MD.0000000000010417

**Published:** 2018-04-27

**Authors:** Zhenhua Xing, Junyu Pei, Liang Tang, Xinqun Hu

**Affiliations:** Department of Cardiovascular Medicine, The Second Xiangya Hospital, Central South University, Changsha, Hunan, China.

**Keywords:** coronary collateral circulation, meta-analysis, protocol, traditional risk factor

## Abstract

**Background:**

Well-developed coronary collateral circulation usually results in fewer infarct size, improved cardiac function, and fewer mortality. Traditional coronary risk factors (diabetes, hypertension, and smoking) have some effects on coronary collateral circulation. However, the association between these risk factors and coronary collateral circulation are controversial. Given the confusing evidences regarding traditional cardiovascular risk factors on coronary collateral circulation, we performed this meta-analysis protocol to investigate the relationship between traditional risk factors of coronary artery disease and coronary collateral circulation.

**Methods:**

MEDINE, EMBASE, and Science Citation Index will be searched to identify relevant studies. The primary outcomes of this meta-analysis are well-developed coronary collateral circulation. Meta-analysis was performed to calculate the odds ratio (OR) and 95% confidence interval (CI) of traditional coronary risk factors (diabetes, smoking, hypertriton). Pooled ORs were computed as the Mantel–Haenszel-weighted average of the ORs for all included studies. Sensitivity analysis, quality assessment, publication bias analysis, and the Grading of Recommendations Assessment, Development and Evaluation approach (GRADE) will be performed to ensure the reliability of our results.

**Results:**

This study will provide a high-quality synthesis of current evidence of traditional risk factors on collateral circulation.

**Conclusion:**

This conclusion of our systematic review and meta-analysis will provide evidence to judge whether traditional risk factors affects coronary collateral circulation.

Ethics and dissemination: Ethical approval is not required because our systematic review and meta-analysis will be based on published data without interventions on patients. The findings of this study will be published in a peer-reviewed journal.

## 1 Introduction

An increasing number of people in developing countries are under traditional cardiovascular risk factors such as smoking, hypertension and diabetes mellitus, et al, which may result in high rates of cardiovascular disease.^[1]^ However, smokers have been shown to have lower mortality after acute myocardial infaction (AMI) compared with nonsmokers.^[2]^ Although this so-called smokers’ paradox has been attributed to the younger age, lower co-morbidity, more aggressive treatment and lower risk profile of the smoker,^[2]^ well-developed coronary collateral circulation may be another explanation. Some investigators have found that smoking was associated with well-developed coronary collateral circulation which might alleviate myocardial ischemia and necrosis when epicardial coronary arteries were occluded.^[3,4]^ Well-developed coronary collateral circulation usually results in fewer infarct size, improved cardiac function, and fewer mortality.^[5–7]^ Smoking improves coronary collateral circulation which may partial explain the so-called smokers’ paradox. But some researchers found opposite evidence that smoking caused rarefaction of coronary collateral circulation.^[8]^

Except from smoking, other cardiovascular risk factors such as hypertension, diabetes mellites also have some impacts on coronary collateral circulation.^[3]^ Yetkin et al^[9]^ found that diabetes mellitus was an independent factor for rarefaction of coronary collateral circulation. However, the study by Niccoli et al^[10]^ found that patients with diabetes mellitus had better collateral development compared with nondiabetic patients. Although, the association between these risk factors and coronary collateral circulation is controversial, these risk factors do have some effects on collateral on coronary collateral circulation. Given the confusing evidences between traditional cardiovascular risk factors and coronary collateral circulation, we performed this meta-analysis protocol to investigate the relationship between traditional risk factors of coronary artery disease and coronary collateral circulation.

## 2 Methods

This systematic review and meta-analysis protocol has been registered with the International Prospective Register of Systematic Reviews (CRD42018087821). This protocol is reported following the Preferred Reporting Items for Systematic Review and Meta-Analysis Protocols (PRISMA) guidance.^[11]^ We conducted a review of traditional coronary artery disease risk factors and coronary collateral circulation by systematically searching for relevant studies in which traditional risk factors (smoking, diabetes, and hypertension) was all or part of the exposure of interest or where traditional risk factors had been part of a subgroup analysis.

## 3 Inclusion criteria for study selection

### 3.1 Studies

Any case–control study was included in our meta-analysis if the extent of coronary collateral circulation of each patient could be extracted according to different traditional risk factors (smoking, hypertension, and diabetes).

### 3.2 Participants

Studies that had patients with chronic total occlusion (CTO) of the main branches of the coronary arteries were included. Ideally, CTO in the included studies should be defined as a totally occluded segment with Thrombolysis in Myocardial Infarction flow grade 0 and an estimated duration of at least 3 months.^[12]^ But we will include those studies did not define CTO exactly. Studies that included patients with severe stenosis (e.g., ≥75% or ≥95%) were excluded.

### 3.3 Exposure factors

Traditional coronary risk factors such as smoking, diabetes, and hypertension were defined as exposure factors. Patients were divided into 2 groups according to patients with or without traditional risk factors (smoking or not smoking, with diabetes or without diabetes, with hypertension or without hypertension).

### 3.4 Outcome measures

The primary outcomes of this meta-analysis are well-developed coronary collateral circulation. The definition of collateral circulation is Rentrop scoring system: 0= no collateral vessels; 1= thread-like, poorly opacified collaterals without visualization of the epicardial artery; 2 = partial filling of the epicardial artery via collateral channels; 3 = complete filling of the epicardial segment of the artery via collateral channels.^[13]^ Well-developed coronary collateral circulation was defined as Rentrop score 2 or 3.

### 3.5 Search strategy

The databases will be searched to obtain relevant studies included MEDINE, EMBASE, Science Citation Index before January 1, 2018. Language is restricted to English. We will use keywords, MeSH term searches and Emtree to find relevant studies. We also will search the reference lists of relevant studies and reviews, editorials, and letters, together with related conference abstracts to identify further articles.

## 4 Data selection, and analysis

### 4.1 Selection of studies

**Figure 1:**
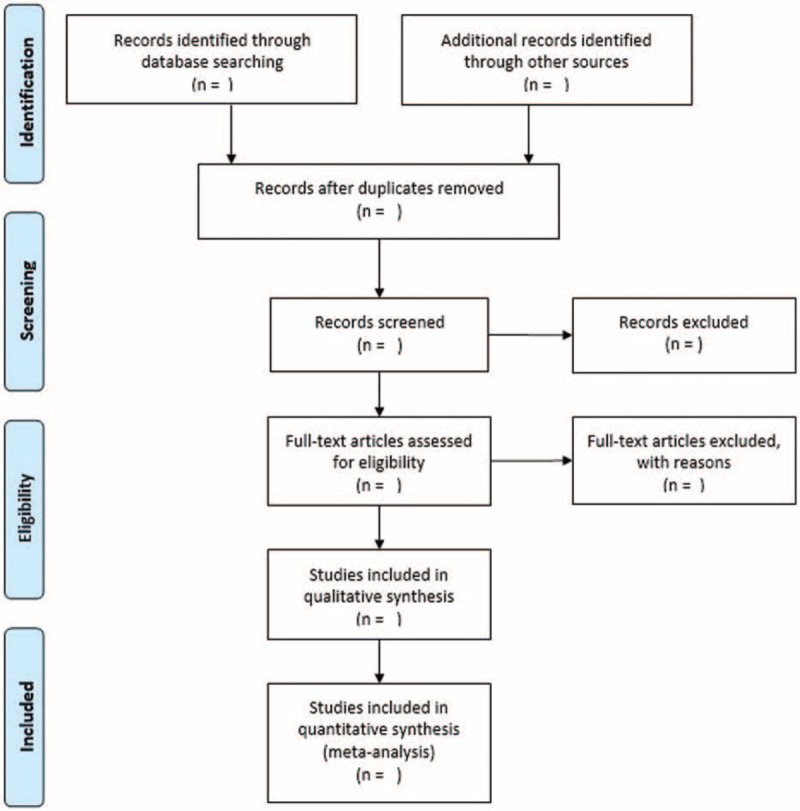
Flow diagram of literature searched for meta-analysis.

Relevant studies were searched by 2 independent investigators (JYP and ZHX). We use Endnote (Thompson ISI ResearchSoft, Philadelphia) to manage relevant articles and remove duplicated articles. Disagreements were resolved by consensus or a third investigator (XQH). The process of study search strategy will be shown in a PRISMA-compliant flow chart (Fig. 1).

### 4.2 Data extraction and management

We abstracted the following data from the selected articles: study characteristics (e.g., first author, publication date, country, et al); characteristics of included participants (e.g., age, sex, smoking, diabetes, hypertension, et al); definition of coronary collateral circulation; and risk factors of coronary collateral circulation. All these data were extracted to a prepiloted, standardized excel sheet. When relevant data were missing, study authors were contacted by e-mail, before excluding the reference for inaccessibility of data.

### 4.3 Assessment of risk of bias in included studies

We assessed the methodological quality of included studies based on Newcastle–Ottawa Scale (NOS) for quality of case–control and cohort studies.^[14]^ A maximum of 2 stars can be given for comparability. A star system of the NOS (range, 0–9) has been performed for the evaluation (Table 1).

**Table 1:**
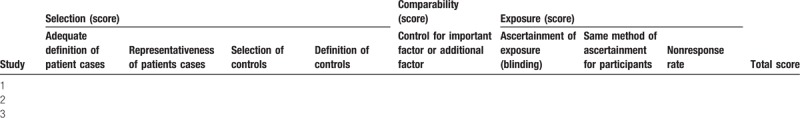
Quality assessments of studies included in these meta-analyses.

### 4.4 Data analysis

Meta-analysis will be performed to calculate the odds ratio (OR) and 95% confidence interval (CI) of traditional coronary risk factors (diabetes, smoking, and hypertriton). Pooled ORs are computed as the Mantel–Haenszel-weighted average of the ORs for all included studies. Since the true treatment effect of various postconditioning protocols may have varied among the included trials, the random-effects model is used in the analysis. Statistical heterogeneity among the trial-specific ORs is checked and quantified by the *I*^2^ statistic, and a *P*-value ≤.05 is considered statistical significant.

If quantitative analysis is not appropriate, we will just perform a narrative, qualitative summary and the results will be presented with text and tables.

### 4.5 Sensitivity analysis

Sensitivity analysis will be performed to assess the contribution of each study to the pooled estimation by excluding one trial at a time and recalculating the pooled ORs estimation for the remaining studies. Both random efforts and fixed-effort model will be used both.

### 4.6 Publication bias

In order to study publication bias on our meta-analysis, publication bias will be qualitatively analyzed by funnel plots as well as qualitatively analyzed by Egger's tests.^[15,16]^ If publication bias does exist, we use “trim and fill” method to analyze publication bias on our outcomes.^[17]^

### 4.7 Confidence in cumulative evidence

The overall evidence then assessed by the Grading of Recommendations, Assessment, Development, and Evaluation (GRADE) approach.^[18]^ This approach will assess the risk of bias, directness of evidence, precision of estimate, heterogeneity, publication bias, effect size, and plausible explanation of the confounding or bias. The quality of evidence will be listed as high, moderate, low, or very low. We will perform this analysis using GRADEpro online software.

## 5 Discussion

Currently, the association between traditional coronary artery disease risk factors and coronary collateral circulation is uncertain. Contradictory evidences and opinions continue to surface. Therefore, it is necessary to perform a high quality systemic review and meta-analysis, in which our rigorous approach will provide a solid evidence for these issues. To our knowledge, this will be the first systematic review and meta-analysis using data of case–control studies to ensure the association between traditional risk factors and collateral circulation for patients with CTO.

## Author contributions

**Conceptualization:** Zhenhua Xing, Xinqun Hu.

**Data curation:** Zhenhua Xing.

**Formal analysis:** Zhenhua Xing.

**Writing – original draft:** Junyu Pei.

**Writing – review & editing:** Liang Tang.
